# Stimfit: quantifying electrophysiological data with Python

**DOI:** 10.3389/fninf.2014.00016

**Published:** 2014-02-21

**Authors:** Segundo J. Guzman, Alois Schlögl, Christoph Schmidt-Hieber

**Affiliations:** ^1^Institute of Science and Technology AustriaKlosterneuburg, Austria; ^2^Wolfson Institute for Biomedical Research, University College LondonLondon, UK; ^3^Department of Neuroscience, Physiology and Pharmacology, University College LondonLondon, UK

**Keywords:** electrophysiology, patch-clamp, data analysis, biosignal data formats, free software, C++, Python

## Abstract

Intracellular electrophysiological recordings provide crucial insights into elementary neuronal signals such as action potentials and synaptic currents. Analyzing and interpreting these signals is essential for a quantitative understanding of neuronal information processing, and requires both fast data visualization and ready access to complex analysis routines. To achieve this goal, we have developed Stimfit, a free software package for cellular neurophysiology with a Python scripting interface and a built-in Python shell. The program supports most standard file formats for cellular neurophysiology and other biomedical signals through the Biosig library. To quantify and interpret the activity of single neurons and communication between neurons, the program includes algorithms to characterize the kinetics of presynaptic action potentials and postsynaptic currents, estimate latencies between pre- and postsynaptic events, and detect spontaneously occurring events. We validate and benchmark these algorithms, give estimation errors, and provide sample use cases, showing that Stimfit represents an efficient, accessible and extensible way to accurately analyze and interpret neuronal signals.

## 1. Introduction

Neurons communicate with each other in a precisely timed, carefully orchestrated and widely tunable process termed synaptic transmission. The critical steps of this process include action potential generation in the presynaptic neuron, neurotransmitter release from the presynaptic terminal, and integration of synaptic inputs in the postsynaptic cell. To understand how information is processed in the brain, it is essential to accurately measure and reproducibly quantify these elementary steps of neuronal communication.

Intracellular patch-clamp or sharp microelectrode recordings provide insight into this neural communication process with unmatched accuracy, resolving membrane potential at the microvolt level with microsecond precision in cell cultures, acute brain slices, anesthetized and awake animals. These techniques can be applied to a single neuron to study its sub- and suprathreshold activity. Alternatively, simultaneous recordings from multiple neurons can be used to directly measure synaptic interactions between neurons. Finally, subcellular axonal and dendritic recordings assess the propagation of activity within a neuron. Data from such intracellular recordings are typically stored as repetitive epoch-like events (“sweeps”) that may be composed of millions of sampling points. Efficient analysis and interpretation of the resulting large datasets require user-controlled fast visualization of recordings, simple selection of relevant sweeps, and straightforward application of analysis routines to single or multiple sweeps.

Given the large variety of experimental questions and approaches in cellular neuroscience, flexibility and extensibility through user customization are fundamental requirements for a data analysis environment. A custom scripting language with an interactive command-line environment represents a common solution but it generally lacks practical use outside of the context of the specific application. In contrast, general purpose programing languages like Python (van Rossum, 2007) give access to supplementary scientific libraries, and provide tools to assist with additional aspects of the analysis, such as storage, organization and sharing of analysis results. Moreover, by fully relying on free and open-source software, reproducibility of analysis routines can be ensured across different systems and platforms (Peng, 2011).

We here present Stimfit, a free analysis environment for cellular neurophysiology. We describe its workflow that utilizes a standard desktop application with oscilloscope-like elements, and show how a Python scripting interface and a built-in Python shell can be used for customizing and extending the program functionality. We then present and validate analysis algorithms and routines that Stimfit uses to quantify neuronal signaling and communication, and provide sample use cases to illustrate the program functionality.

## 2. Analysis workflow

**Figure 1:**
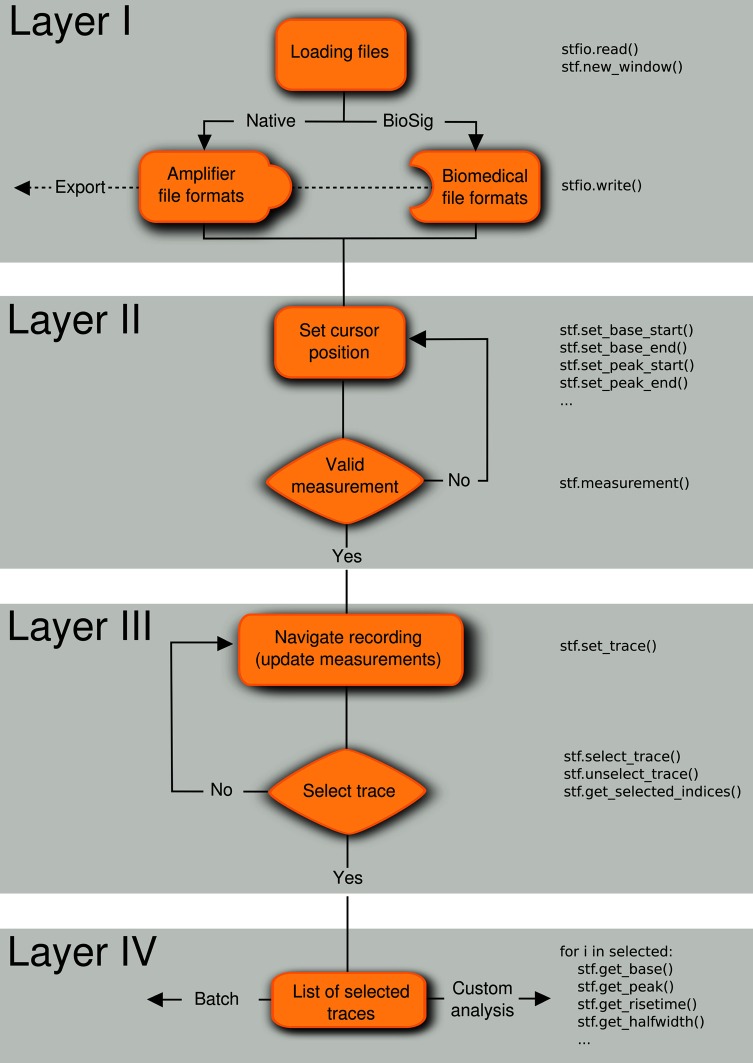
**Analysis workflow**. A four-layered analysis workflow that includes (I) loading/exporting the recordings from/to various file formats, (II) setting the location of the cursors in the regions of interest, (III) navigating through the traces to select experiments matching inclusion criteria, and (IV) applying measurements to the list of selected traces. The code in the right part of the flow diagram shows how these operations can be performed from the embedded Python shell. Note that the layers can be applied in any order, for example first select traces (layer III) and last save selected traces in a file (layer I).

We developed a natural analysis workflow to efficiently select and analyze representative electrophysiological signals that are acquired in consecutive traces (“sweeps”). Our goal was to design a software with a general desktop application interface (i.e., with standard menus and mouse interaction) that incorporates concepts that are familiar to neurophysiologists, such as the presence of cursors in digital oscilloscopes. We devised the analysis workflow in four conceptually distinct layers (Figure 1).

### 2.1. Layer I: file import and export

Electrophysiological data are commonly acquired using an integrated commercial recording system comprising an amplifier, an analog-to-digital converter (ADC) and a compatible software package for controlling and storing the recordings. Usually, each vendor uses its own data format, limiting interoperability between platforms and complicating data exchange between scientists. Stimfit overcomes this problem by supporting a large range of data formats, including the most common file types in cellular neurophysiology (ABF/ATF, AXG, CFS, Heka/Patchmaster, see Table 1). Data can be exported to CFS, GDF, HDF5 or IBW formats for further processing with other software packages.

**Table 1:** **List of supported file formats**.

**File type**	**Brief description**	**Read**	**Write**
ABF	Axon binary file format 1 (pClamp versions 6–9)	Yes^+^	No
ABF2	Axon binary file format 2 (pClamp versions 10+)	Yes	No
ATF	Axon text file format	Yes	Yes
AXGX/AXGD	Axograph X file format	Yes	No
CFS	Cambridge electronic devices filing system	YesYes^+^	Yes
GDF	General dataformat for biosignals	Yes^*^	Yes^*^
HDF5	Hierarchical data format 5	Yes	Yes
HEKA	HEKA binary file format	YesYes^+^	No
IBW	Wavemetrics Igor binary waves	Yes^*^	Yes

*Only formats relevant for cellular physiology are listed*.

(*) indicates support through Biosig,

(+) *indicates improved support through Biosig*.

As an additional file handling backend, the Biosig library has been included, adding support for more than 30 additional file formats and providing a common software interface to access them (Vidaurre et al., 2011). Biosig also supplies an automated file format identification, reducing the need for the user to select the correct import filter. It natively supports the “General Data Format for biomedical signals” (GDF, Schlögl, 2013), which combines features of various standards in biosignal data formats in a single format, in the hope of reducing proliferation of mutually incompatible data formats. With Biosig, signals from various native formats, such as fluorescence data, can be converted to GDF for further analysis with Stimfit or other common analysis platforms, like MATLAB/Octave.

To access data stored in common electrophysiology formats from Python without running Stimfit, a standalone Python module (stfio) has been developed. An accompanying Python module, stfio_plot, includes functions that replace coordinate axes with scalebars in plots generated with the matplotlib library, as is customary when displaying electrophysiological traces.

### 2.2. Layer II: positioning of measurement cursors

After loading a recording, individual traces of a data set are presented as on an oscilloscope, with pairs of vertical cursors delimiting regions of interest. Once the user has chosen the cursor positions (baseline, peak, decay, and latency), measurements are performed within the cursor regions of the currently displayed trace.

### 2.3. Layer III: trace selection

During navigation through a file, data are displayed using a fast algorithm (described below), and all measurements are updated so that quantitative criteria can be used to select traces for further analysis. If required, selected traces can be concatenated to a single uninterrupted trace or visualized in a separate window.

### 2.4. Layer IV: analysis on selected traces

Finally, a set of analyses can be applied to the list of selected traces. Basic analysis routines include baseline substraction, averaging across traces, filtering, etc. Moreover, traces can be fitted to common models in neuroscience (alpha synapse function, multiexponentials, etc.), and spontaneous synaptic events can be extracted from the traces.

Every layer of the workflow can be executed independently or in a different sequence. For example it is possible to first select traces (layer III) and then save the selection to a file (layer I). All layers can be controlled from a Python shell that is embedded into Stimfit. This permits both a direct interaction with the program (e.g., control cursor location, return measurements) and access to the data using the stf module. An example script is given in Supplementary Listing 1.

## 3. Results

### 3.1. Fast trace visualization

**Figure 2:**
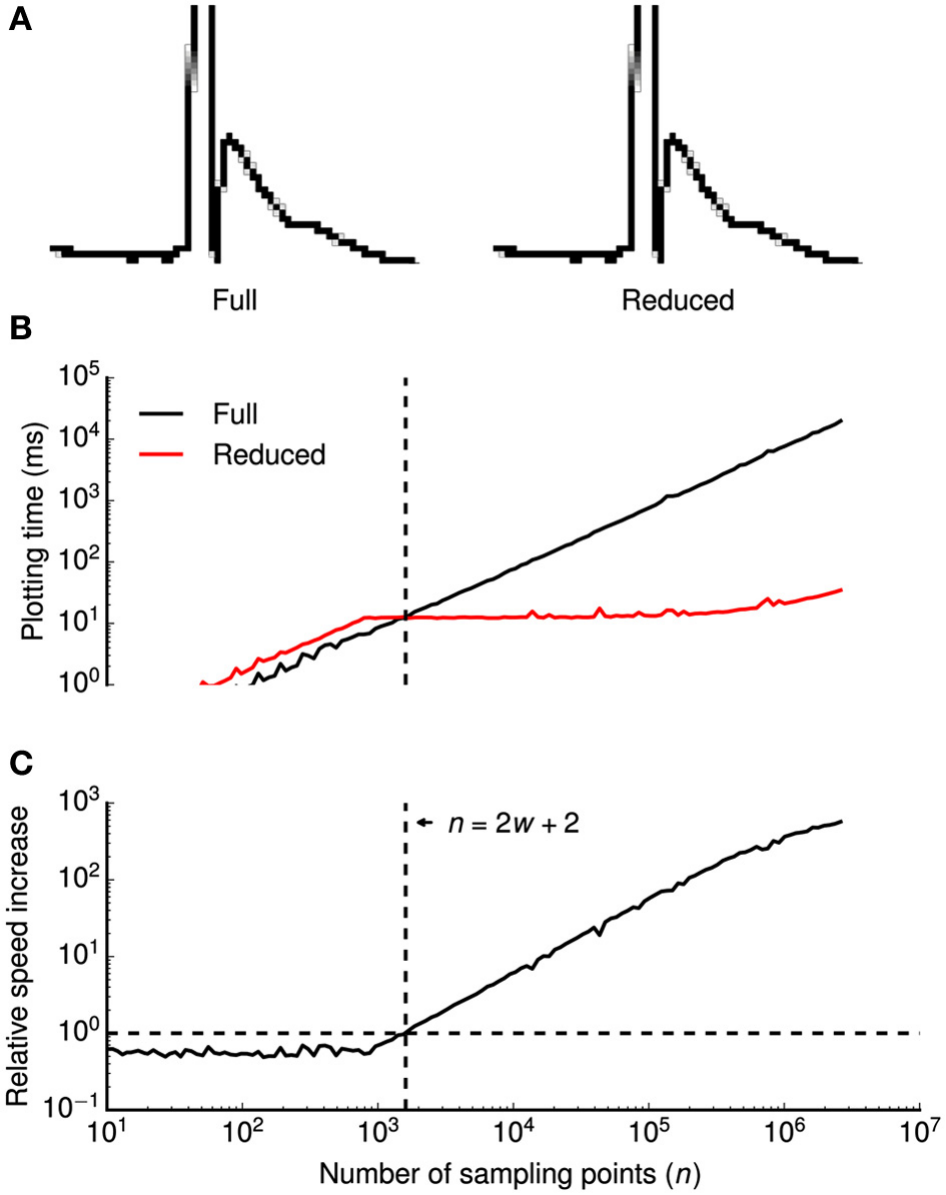
**Data reduction benchmark**. A data reduction algorithm provides up to ~500-fold speed increase without visual information loss. **(A)** High-magnification view of individual pixels when all consecutive points in a time series (a clipped action potential) are connected by lines (left) or when a data reduction algorithm is applied (right). While the algorithm reduces the amount of plotted lines by a factor of ~16 in this example, all details of the time series are visually preserved. Gray shaded pixels are caused by the anti-aliasing algorithm of the line drawing back-end. **(B,C)** Plotting times (**B**) and relative speed increase by the data reduction algorithm (**C**) were plotted double-logarithmically against the number of sampling points *n* of the time series. Relative speed increase was computed as the ratio of plotting times without and with data reduction. In **(B)**, plotting times are shown with (red) and without (black) data reduction. Vertical dashed lines in **(B,C)** denote the minimum number of sampling points *n* that is required for the algorithm to be beneficial (speed increase >1.0), indicated by a horizontal dashed line in **(C)** at a horizontal screen resolution *w* (800 pixels in this example). Plotting times were measured for a 800 × 600 pixels window running on OS X.

To efficiently visualize data sampled with high frequencies at uniform time intervals, we devised a down-sampling algorithm that minimizes the number of plotted lines while preserving the visual information from the original time series (Figure 2A).

The basic principle of the algorithm is to avoid redundant line drawings within vertical pixel columns that will occur if the number of sampling points *n* (spaced equally in time) exceeds the horizontal screen resolution *w*. Without data reduction, visualizing the full time series requires *n* − 1 line drawings between subsequent samples. Within each pixel column, *n*/*w* − 1 vertical lines are plotted, covering all pixels between the vertical minimum *y*_min_ and the maximum *y*_max_ of the pixel column. As drawing *n*/*w* − 1 lines is more expensive than finding the extreme of *n*/*w* points, our algorithm replaces these lines by a single line between *y*_min_ and *y*_max_. It then proceeds to the next pixel column by connecting the last sample of the current column with the first sample of the subsequent column.

In summary, two lines will be drawn per pixel column, that is a total of 2*w* − 1 lines. Notably, the number of lines is independent of the number of sampling points *n*. Since the algorithm saves *n* − 2*w* − 2 line drawings, it is only beneficial if *n* > 2*w* + 2. When displaying a sweep of 2.5 · 10^6^ data points (e.g., 50 s sampled at 50 kHz), our algorithm accelerates plotting up to ~500-fold from ~18 s to ~35 ms (Figures 2B,C).

### 3.2. Principal measurements

**Figure 3:**
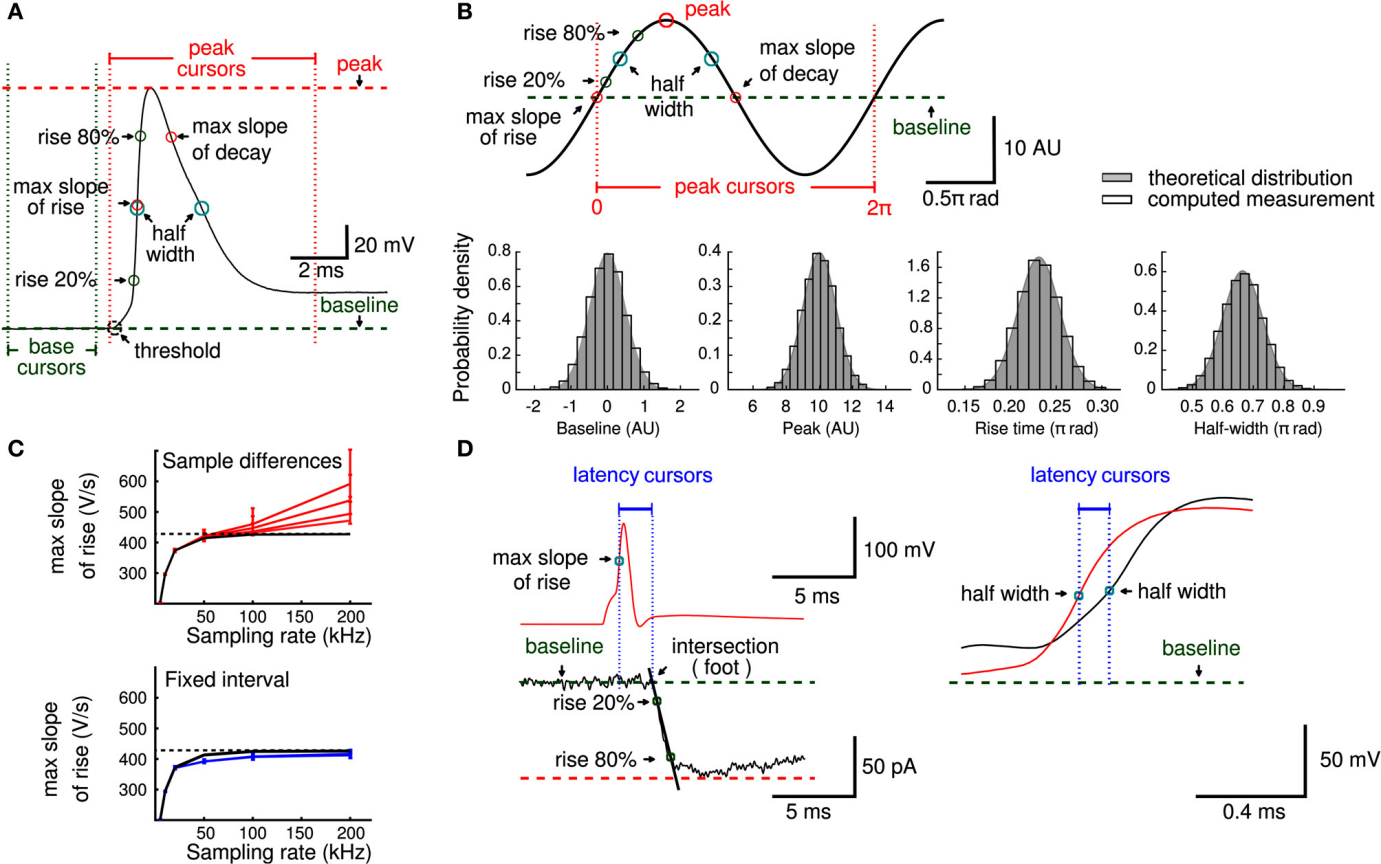
**Principal measurements. (A)** An action potential showing baseline (green) and peak (red) cursors that delimit the principal measurements obtained in layer II of the analysis workflow. **(B)** An example of a trace used to provide analytical solutions to validate principal measurements (peak 10 arbitrary units, rise time 0.23 π rad, half duration 0.66 π rad, max slope 1 and max decay −1). Histograms of baseline, peak, rise time and half duration measurements (open bars) show estimated measurements together with the theoretical distribution used to generate the analytical values (gray). **(C)** The error to estimate the maximal slope depends on sampling rate and measurement noise. The black line is the expected maximal slope when sampling noise free data, in red are the slope values and its 5 and 95 percentiles obtained when computed slopes from adjacent sampling differences at different noise levels (impedance noise of 10, 20, 50, and 100 MΩ, respectively). Blue lines indicate the slope values calculated from the traces in red, but with a fixed interval (temporal window of 50 μs). Note smaller confidence intervals and lower dependency on the sampling rate. Dashed line indicates the maximal slope value (428.1 V/s) used as reference. **(D)** Parameters of physiological relevance, like latencies between an action potential (red) and a postsynaptic response (black) or between axonal (red) and somatic (black) action potentials can be computed from principal measurements.

We first sought to validate the principal measurements that Stimfit performs, including baseline, peak, rise time, half duration (full width at half-maximal amplitude), and maximal slopes of rise and decay of an electrophysiological signal (Table 2). We restrict the validation to these principal measurements, since all other standard measurements (e.g., baseline standard deviation, threshold crossing time, latencies, etc.) are derived from them. To validate our implementation of the principal measurements, we ensured that the measured values did not differ from known values obtained from idealized traces. We generated idealized traces based on common functions (like sine or exponential functions) that allowed us to analytically obtain expected measurement values. To mimic the ranges of values observed under experimental conditions, we generated 10,000 data sets by multiplying function parameters with random numbers drawn from a normal distribution (see an example in Figure 3B). All measurements passed the validation (e.g., returned the expected analytic values), showing that they are accurate and robust. In addition, to evaluate the computation time of the measurements we determined the average time for 10 validations with normally distributed parameters. The execution times were small (see Table 2) as tested on a standard computer employed for such analysis. Because routines were fast and accurate, we decided to make them accessible to Python in the stf module using the wrapper generator SWIG (Beazley, 2003).

**Table 2:** **Description of principal measurements**.

**Measurement**	**Return values (units)**	Execution time^*^ (ms)
Baseline	Average value (*y* units)	2626 ± 25
Threshold crossing	Value where the slope exceeds a predefined rate value (*y* units)	2219 ± 5
Peak value	Local maxima/minima (*y* units)	705 ± 10
Rise time	Time difference between peak fractions (e.g., 20% and 80%) (*x* units)	1115 ± 4
Half duration	Full width at half-maximal peak amplitude (*x* units)	1155 ± 6
Slope of rise	Maximal positive slope on the rising phase of the peak (*y*/*x* units)	757 ± 3
Slope of decay	Maximal negative slope on the decay phase of the peak (*y*/*x* units)	613 ± 6

* *Average time of 10 validations of the measurement with 10,000 randomly generated signals. Data are expressed as mean ± SD*.

For measurements that involved the computation of a time derivative, we designed a strategy that minimizes the effect of instrumental error (e.g., quantization, amplifier noise) that occurs upon acquisition at high sampling rates. A common approach to evaluate slopes (e.g., maximal slope of rise of an action potential) is to compute the signal difference between two adjacent sampling points. While slope estimation with this method may be accurate for low-noise recordings acquired under appropriate filtering (Nyquist conditions), slopes can be overestimated due to the noise if the signals are acquired at very high sampling rates (Figure 3C). This is because the temporal derivatives of the noise components (e.g., impedance, amplifier and quantization noise) increase with the sampling rate. Computations on simulated data showed that not only the estimation of the maximal slope is affected, but also the confidence interval of the slope estimate increases (i.e., becomes less accurate, see Figure 3C). We therefore decided to compute the derivatives at a fixed time interval of 50 μs. This yielded more accurate slope estimates and reduced the dependency of the estimate on the sampling rate and noise level (Figure 3C).

As the measurements were reliable, we used them to compute additional parameters of physiological relevance (Figure 3D), such as latencies between action potentials (calculated between peaks or between half-maximal amplitudes) or synaptic latencies. Synaptic latency has been defined as “the time interval between the peak of the inward current through the synaptic membrane and commencement of inward current through the postsynaptic membrane” (Katz and Miledi, 1965). The maximal inward current during an action potential is expected to flow at the time of maximal slope during the rising phase. The commencement (sometimes called “foot”) of the postsynaptic current can robustly be estimated from the extrapolated intersection of the baseline with a line through the two points of time when the current is 20% and 80% of the peak current (Jonas et al., 1993).

### 3.3. Model fitting

**Figure 4:**
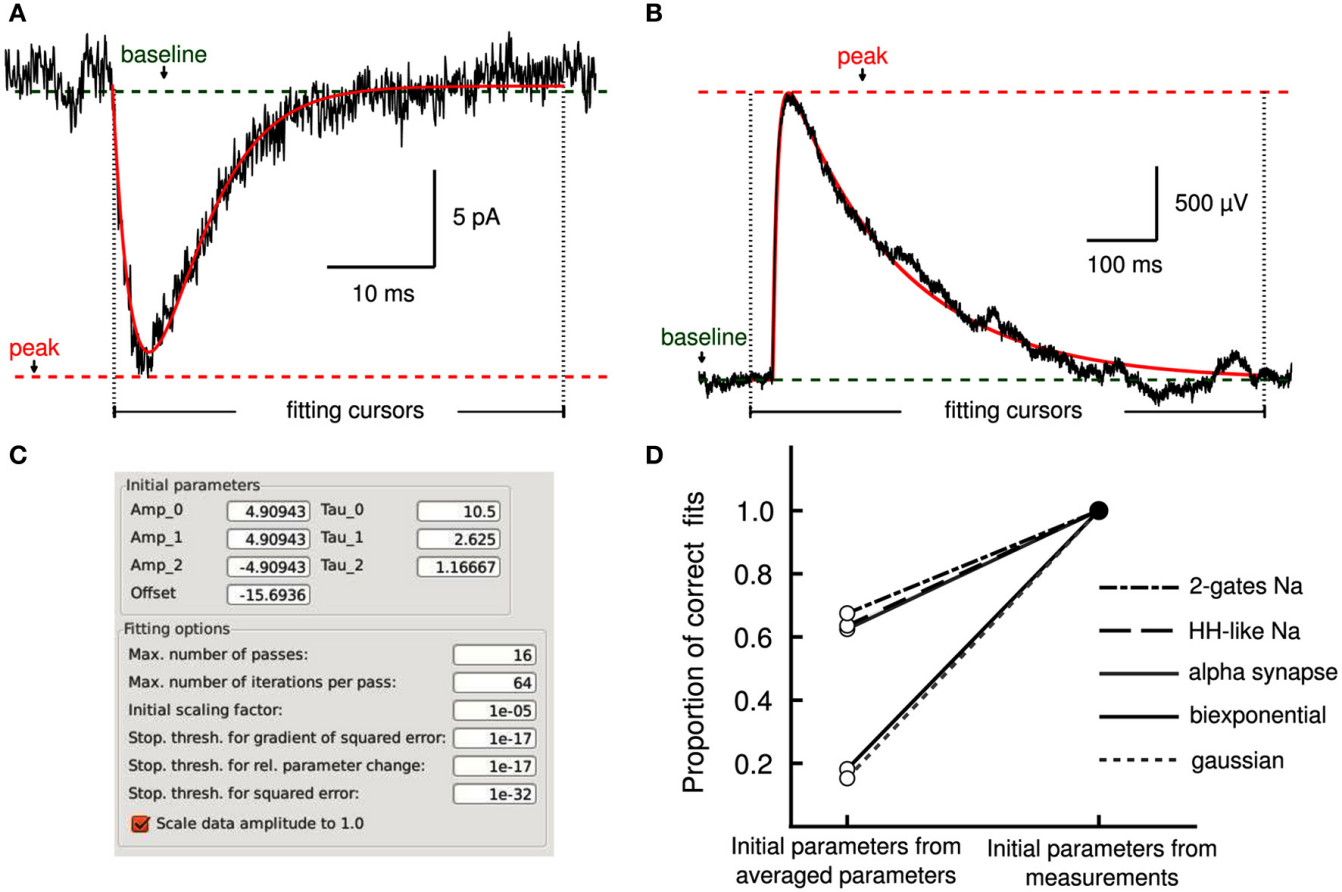
**Model fitting by least-squares optimization. (A)** Average excitatory postsynaptic current (EPSC, in black) together with a fit to an alpha synapse model (red) with a rate of 3.4 ms and amplitude of 13.7 pA. **(B)** A single unitary excitatory postsynaptic potential (EPSP, in black) was fitted to a bi-exponential function (red) with time constants of 7.1 and 161.7 ms, respectively. Cursors delimit the fitting regions. Measurements performed by Stimfit (e.g., baseline, peak) provide appropriate initial conditions to the fitting algorithm. **(C)** User interface allowing detailed manipulation of both initial parameters to the fitting model, and fitting options of the fitting algorithm. **(D)** Without the aid of the measurements to obtain adequate initial estimates, only a proportion of fits converges on the correct values (an SSE between trace and fit >0.001 is considered as a failure). If initial estimates are derived from the measurements (baseline, peak, rise time, and half-width), the fits converge correctly in all cases.

Describing the kinetics of electrophysiological signals often requires fitting observations to various models. We used an implementation of the Levenberg–Marquardt (LM) least-squares optimization algorithm (Lourakis, 2004) for model fitting and adapted it to the analysis workflow. This was achieved by allowing the selection of regions of interest in the data with fitting cursors (Layer II, Figures 4A,B) to guide the fitting operation along the standard workflow (Layer III and IV). Settings for the fitting algorithm, such as stopping conditions, are made accessible to the user (Figure 4C).

Convergence of a fit can be improved by choosing appropriate initial parameters. In some cases, the user may be able to provide adequate initial parameters (Figure 4C) close to the optimum. However, an automated execution of the fitting procedure, including the choice of initial parameters, is often desirable, in particular when operating on large data sets. We devised several strategies to automatically estimate appropriate initial parameters. For exponential models, we obtain parameters from a linear regression on logarithmically transformed data. For other models, we estimate the initial parameters based on the values returned by the principal measurements described in section 3.2 (i.e., peak, baseline, rise time and half duration). For example, the rise time of a current transient can be used to estimate the activation time constant for a Hodgkin–Huxley model. To evaluate our approach to initializing parameters, we fitted idealized traces that we generated from model functions using a range of known values for each function parameter. In all cases, our approach resulted in a convergence to the correct solution (i.e., the global minimum of the least-squares merit function; see Table 3). In contrast, when we initialized each function parameter with the average of all values used to generate idealized traces, we found that some fits did not converge (i.e., sum of squared errors (SSE) >0.001 for a single trace; see Table 3). Thus, our approach to initialize parameters from the measurements provided the conditions necessary to perform a correct fit (see Figure 4D) without user interaction. Optionally, data can be rescaled to have range [0, 1] in *x* (typically time) and *y* before fitting to improve convergence when parameters are badly scaled (Dennis and Schnabel, 1996).

**Table 3:** **Impact of initial estimates on Levenberg–Marquardt fit results**.

**Model**	SSE^*^	**Unsuccessful fits (%)**	SSE^*^	**Unsuccessful fits (%)**	**Traces tested**
	**Estimates from measurements**		**Estimates from average**		
Two-gated Na^+^ conductance	6.7 × 10^−4^	0	5.0 × 10^4^	32.5	5120
Hodgkin–Huxley Na^+^ conductance	6.6 × 10^−4^	0	6.1 × 10^4^	36.4	5120
Alpha synapse	8.7 × 10^−28^	0	5.2 × 10^5^	37.5	6240
Bi-exponential with delay	1.2 × 10^−12^	0	3.6 × 10^2^	81.8	10,500
Gaussian function	2.8 × 10^−28^	0	5.1 × 10^1^	84.7	6860

* *Sum of squared errors between the idealized trace and the best fit averaged over the number of traces tested*.

We validated the fitting algorithms similarly as we did for the principal measurements by creating data sets from all our available models with combinations of known parameters. All models were fitted by the algorithm with a tolerance level of a single sampling point for the unknowns.

The fitting procedure is made available from the embedded Python shell. An example script is given in Supplementary Listing 2.

### 3.4. Event detection methods

**Figure 5:**
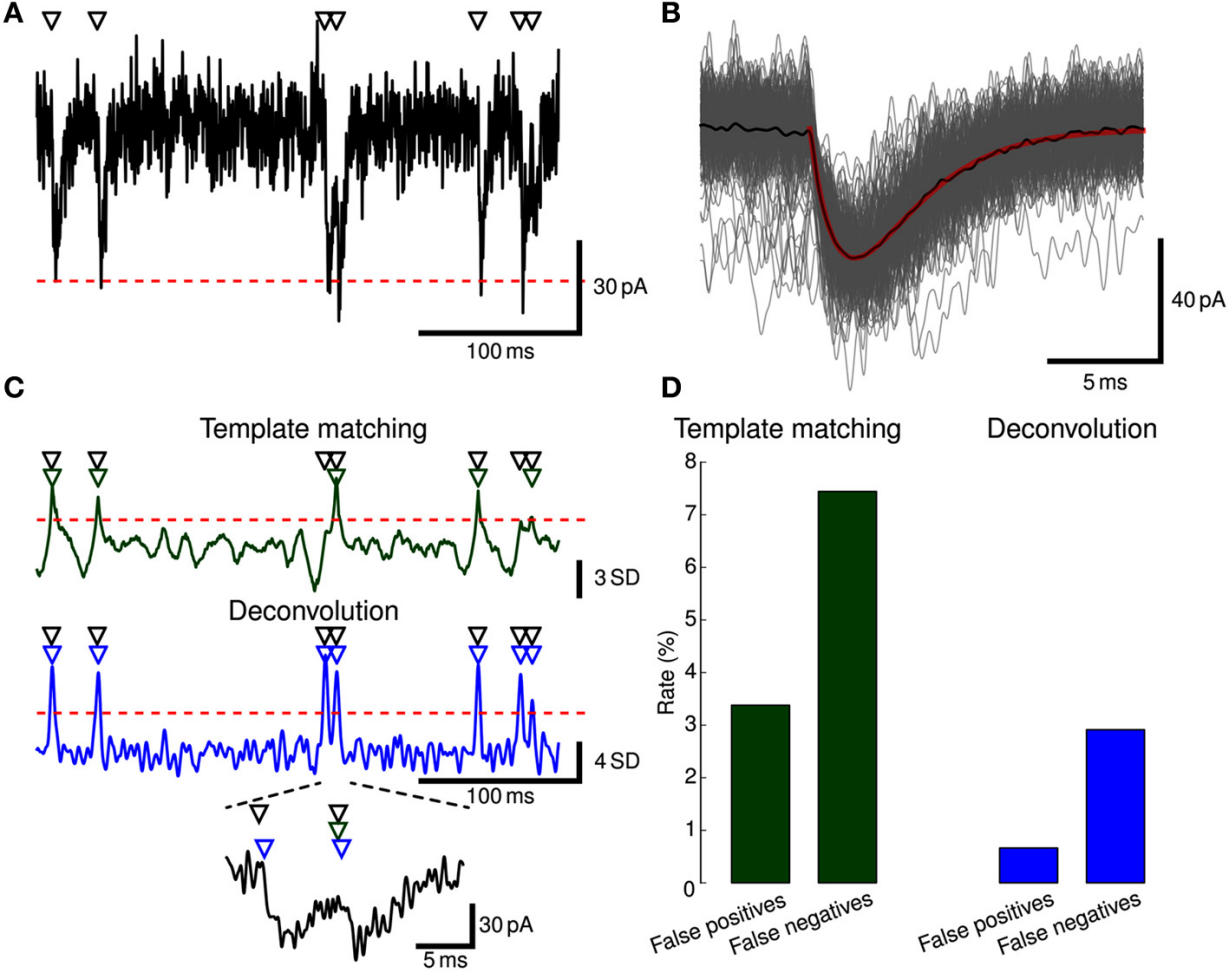
**Event detection. (A)** EPSCs were simulated in a ball-and-stick model with NEURON. Onset times of synaptic conductance changes (triangles at top) were simulated as a Poisson process to yield a mean EPSC frequency of 5 Hz. Colored noise was added as low-pass filtered white noise (*f*_*c*_ = 1.0 kHz). Red dashed line indicates mean −5 SD. Only a subset of the full simulation (60 s duration) is shown. **(B)** To generate a template waveform, we first extracted data periods surrounding negative peaks below mean −5 SD (gray traces). The peak-aligned average of these data periods (black trace) was then fitted to a bi-exponential function (red trace) serving as a template. **(C)** Detection criterion according to template matching (green) and deconvolution (blue) for the EPSCs shown in **(A)** computed with Stimfit. Events were detected when the criteria exceeded a threshold of 2.5 SD (template matching) or 4 SD (deconvolution), as indicated by red horizontal dashed lines. Two overlapping EPSCs are shown at the bottom at higher magnification. Detected events are indicated by green (template matching) or blue (deconvolution) triangles above the traces. **(D)** Error rates for the template matching (left, green) and deconvolution (right, blue) algorithms. Thresholds were adjusted to yield a small total number of false positive and negative events.

Stimfit includes template matching and deconvolution algorithms to isolate individual events such as EPSCs or EPSPs from recorded data (Figure 5).

#### 3.4.1. Template matching

A template matching algorithm was implemented as described by Jonas et al. (1993), with some additional details adopted from Clements and Bekkers (1997). The template consists of a waveform *p*(*t*) with a length of *n* sampling points that represents the time course of a typical event. The template is slid over the recorded signal *r*(*t*), and at each sampling point with index *s*, it is multiplied by a scaling factor *m* and an offset *c* is added or subtracted so that the sum of squared errors χ^2^(*t_s_*) between the trace and the template is minimized:

$$\chi^{2}(t_{s})=\sum_{k=1}^{n}{\left[r\left(t_{s+k}\right)-\left(m\cdot p\left(t_{k}\right)+c\right)\right]}^{2}$$

As can be seen from this equation, this amounts to the simple operation of fitting a straight line that relates *p*(*t*) and *r*(*t*) at every sampling point.

Finally, some detection criterion has to be applied to decide whether an event has occurred at a sampling point. Two options are available in Stimfit: Jonas et al. (1993) suggest to use the linear correlation coefficient between the optimally scaled template and the data, whereas Clements and Bekkers (1997) compare the scaling factor with the noise standard deviation.

#### 3.4.2. Deconvolution

A deconvolution-based algorithm was implemented according to Pernía-Andrade et al. (2012). The basic idea is to describe the recorded signal *r*(*t*) as a convolution *h*(*t*) of the time course of event onsets *f*(*t*) with the time course of a typical event *p*(*t*):

$$h(t)=\int_{0}^{t}f\text{​}\left(t-t^{\prime }\right)p\left(t^{\prime }\right)\text{d}t^{\prime },$$

where *f*(*t*) describes event onsets by the Dirac delta function:

$$f(t)=\delta \text{​}\left(t-t_{0}\right)=\{\begin{array}{ll} \infty & \text{for }t\text{ = }t_{\text{0}},\\ 0 & \text{for }t\neq t_{\text{0}}, \end{array},$$

where *t*_0_ is the time point of the onset of an event.

To detect events, an estimate of *f*(*t*) is obtained (*f*′(*t*)) by deconvolving the recorded signal *r*(*t*) from the template time course *p*(*t*). As for the template matching algorithm, a detection criterion needs to be applied. Following Pernía-Andrade et al. (2012), we fit an all-point histogram of *f*′(*t*) with a Gaussian function. The detection threshold is then set as a multiple (typically 4.0–4.5) of the standard deviation of the fitted Gaussian function.

#### 3.4.3. Practical approach to event detection

In practice, the following steps need to be performed to extract events with Stimfit:

1. Extract some exemplary large and isolated events (Figure 5A).
2. Create a template by fitting a function to the average of the exemplary events (Figure 5B).
3. Extract all events with the final template using a low detection criterion threshold (Figure 5C).
4. Use the GUI to eliminate false-positive and add false-negative events (Figure 5D).

#### 3.4.4. Event detection with python

The event detection algorithms are accessible from the Python shell through the function stf.detect_events(). It takes an arbitrary template waveform as input and returns a detection criterion array. Peaks can be extracted from the detection criterion array using stf.peak_detection(). An example script is given in Supplementary Listing 3.

## 4. Conclusions

We have described and validated analysis algorithms for cellular neurophysiology that are available in Stimfit, a free, open-source and cross-platform application. Its focus lies on viewing and analyzing electrophysiological signals obtained with patch-clamp techniques, such as subcellular recordings from dendrites and axons (Kim et al., 2012), whole-cell recordings from individual cells (Schmidt-Hieber et al., 2007; Guzman et al., 2010) or paired recordings from synaptically connected neurons (Eggermann and Jonas, 2011). In addition to measuring parameters of physiological relevance, the program includes detection routines for spontaneous events, and an implementation of the Levenberg–Marquardt algorithm (Lourakis, 2004) to fit the data to standard mathematical functions (single and multiexponentials) and common models in cellular neuroscience. Thereby, both quantification of signals and model testing can be performed within the same analysis platform using an intuitive workflow. Analysis routines employed in Stimfit were devised and validated across several laboratories over many years and have been used in many peer-reviewed publications.

Using the Biosig library as a backend for file I/O (Vidaurre et al., 2011), Stimfit supports several typical formats for biomedical signals, including those most commonly used in cellular electrophysiology. Moreover, Biosig provides native, direct and automated data format recognition that does not require additional programing effort from the user, in line with Stimfit's goal to offer an easily accessible analysis solution.

The analysis algorithms are accessible from a desktop application and from an embedded Python shell. This strategy combines an accessible and efficient analysis environment with the flexibility of a general-purpose scriptable programing language, making the program extensible and customizable through the extensive scientific computing ecosystem that is available for Python (Oliphant, 2007). Python is widely used in neuroscience (Davison et al., 2009) and has been adopted by most popular neural simulation environments, such as NEURON (Hines et al., 2009), NEST (Eppler et al., 2009), and more recently Genesis (Cornelis et al., 2012). Therefore, an analysis environment that relies on Python will also facilitate seamless evaluation and integration of results from computer simulations.

A number of alternative free software packages are available for data analysis in cellular neurophysiology (e.g., OpenElectrophy, Garcia and Fourcaud-Trocmé, 2009; Spyke Viewer, Pröpper and Obermayer, 2013; WinWCP, http://spider.science.strath.ac.uk/sipbs/software_ses.htm; RELACS, http://relacs.sourceforge.net/), some of which include modules for recording data (WinWCP, RELACS). Stimfit is distinct from these packages in that it is cross-platform and Python-scriptable (in contrast to WinWCP and RELACS), specializes on intracellular recordings (in contrast to OpenElectrophy and Spyke Viewer), and features an embedded shell for direct on-line interaction with the graphical user interface (in contrast to OpenElectrophy, WinWCP, and RELACS).

While the program's main focus currently lies on the quantification of single and paired intracellular recordings, we aim to extend this to extracellular and imaging data by joining forces with other projects within the lively Python neuroscience ecosystem. To facilitate interoperability with other Python software tools, we plan to adopt a common, shared object model for representing electrophysiological data in Python, as described by the neo project (http://neuralensemble.org/neo/). Moreover, we aim to develop an interface to OpenElectrophy to improve support for extracellular recordings and interchangeable storage of data and meta-data. The long-term vision is to provide universal, validated and standard free software tools for the analysis of a wide variety of neuroscientific multi-channel time series.

## 5. Material and methods

### 5.1. Software design

The core application is written in C++, making use of several open-source C/C++ libraries (see Table 4). The C++ toolkit library wxWidgets (Smart et al., 2005) was chosen to create a graphical user interface (GUI) providing native controls and utilities for all available platforms (Microsoft Windows, GNU/Linux and Apple Mac OS X). In general, the source code can be compiled with any ANSI/ISO C++ compiler and has been tested with the GNU compiler collection (gcc/g++) for GNU/Linux, Mac OS X, the MinGW-cross-compiler environment (MXE, Grabsch, 2013), as well as with Microsoft Visual C++ 2008 (MSVC2008) for Windows (only 32-bit tested). A 64-bit version for Windows without Python support can be built with MXE (https://github.com/schloegl/mxe.git).

**Table 4:** **List of C++ external libraries**.

**Library**	**Brief description**	**Full program**	**Standalone module**
Biosig	Biosignal file format support	Yes	Optional
Boost	C++ library	Yes	No
FFTW	Fast fourier transform	Yes	No
GTest	Google unit testing framework	Yes	No
HDF5	File format for large data sets	Yes	Yes
LAPACK	Linear algebra package	Yes	No
levmar	Levenberg–Marquardt non-linear regression	Yes	No
Python	Scriptable general programing language	Yes	Yes
wxPython	GUI toolkit for Python	Yes	No
wxWidgets	GUI toolkit for C/C++	Yes	No

Stimfit is available for GNU/Linux through the standard Debian repositories and NeuroDebian (Halchenko and Hanke, 2012), for OS X through the MacPorts repository (https://www.macports.org), and for Windows as a binary installer.

### 5.2. Experimental procedures

In the figures, original traces show whole-cell patch-clamp recordings obtained from acute brain slice preparations of the hippocampus as described in Schmidt-Hieber et al. (2004). In Figure 3A, an action potential is evoked by somatic current injection in a granule cell of the dentate gyrus. Figure 3D shows excitatory postsynaptic currents evoked by an action potential between synaptically connected CA3 pyramidal neurons and simultaneous somatic and axonal recordings of an action potential originated at the mossy fiber axon. In Figures 4A,B excitatory postsynaptic currents and corresponding potentials were evoked by a single presynaptic CA3 neuron via recurrent collateral synapses.

### 5.3. Validation of measurement and fitting algorithms

All validations were executed on a PC with a 2.5 GHz Intel Core i5 CPU running on GNU/Linux. To validate principal measurements, we generated synthetic data from analytic functions. As an example, we used a sine function of the form

g​(x,A,f)=Asin2πfx,

where *x* is the independent variable, and *A* and *f* are amplitude and frequency, respectively. The expected peak measurement will correspond in this case to *A*. The expected 20–80% rise time is (arcsin0.8 − arcsin0.2)*f*, and the expected half width is 2(arcsin1 − arcsin0.5)*f*. For example, if we generate a sine function with frequency 1, the rise time will be 0.23 π radians, and the half duration 0.66 π radians. Finally, the expected maximal slopes of rise and decay will be located where cos2π*fx* = 1 and cos2π*fx* = −1, respectively. For threshold and baseline measurements we employed a mono-exponential function and a Gaussian function, respectively. We then tested 10,000 variations of randomly distributed parameters. Following this approach, the validation was considered successful if the value returned by the measure corresponded to the analytic result with one sampling point accuracy. In addition, results of the principal measurements were checked by re-implementing the algorithms in Python or Octave code that returned identical values for the same data sets.

To quantify the fitting performance to different models (i.e., proportion of correct fits), we generated synthetic traces from the model functions using known parameter combinations (see Table 3). A trace was considered to be successfully fitted if the parameters were correctly retrieved and if the sum of squared errors (SSE) between the trace and the model was below 0.001. Thus, the proportion of fits is the number of traces successfully fitted divided by the total number of traces tested. For validating the fitting algorithm we used a similar strategy that consisted in generating a set of waveforms with known parameters that were fitted to the models.

### 5.4. Evaluation of the slope estimation error

When computing slopes, we used a realistic action potential waveform simulated at an integration interval of 10^−3^ μs that we stored with double precision accuracy. The maximal slope of rise of the waveform was 428.1 V/s. We added thermal noise by applying impedance values from 0 to 100 MΩ to the expression

$$\sqrt{4k_{B}TBZ},$$

where *k*_*B*_ is the Boltzmann constant, *T* the absolute temperature, *B* the corresponding Nyquist frequency and *Z* the impedance. The waveform was down-sampled to frequencies in the range of 5–200 kHz and rounded to 2 V/2^16^ to mimic the quantization noise. Next, we calculated the maximal slope of rise for every sampling rate and thermal noise level. The maximal slope of rise was calculated using the difference between two consecutive sampling points or within a fixed time interval. The interval was fixed to 50 μs for sampling rates of 20 kHz or higher. For sampling rates which are not multiples of that frequency, the interval was fixed to the number of samples closest to 50 μs. Standard deviation and 5 and 95 percentile values were calculated from 100 different noise realizations.

### 5.5. Computer simulations

Simulated traces were generated in NEURON 7.3 with Python 2.7 as interpreter (Hines et al., 2009). To generate the action potential waveform used for the validation of the slope algorithm, a small current injection (100 pA for 2 s) was injected into a single compartment with a specific membrane capacitance of *C*_*m*_ = 1 μF cm^−2^, a leak conductance of 0.1 mS cm^−2^ and active sodium (35 mS cm^−2^) and potassium (9 mS cm^−2^) peak conductances, as described by Wang and Buzsáki (1996).

To validate event detection algorithms, excitatory postsynaptic currents (EPSCs) were generated in a ball-and-stick model with a somatic diameter and length of 20 μm, a dendritic length of 500 μm and a dendritic diameter of 5 μm. Specific membrane capacitance *C*_m_ was 1 μF cm^−2^, specific membrane resistance *R*_m_ was 25 kΩ cm^2^, and specific axial resistivity *R*_a_ was 150 Ω cm. Excitatory synaptic conductance changes had a bi-exponential time course with τ_onset_ = 0.2 ms, τ_decay_ = 2.5 ms, a peak amplitude of 1 nS and a reversal potential of 0 mV. Dendritic locations of synaptic conductance changes were distributed on the dendrite according to a normal distribution with a center at 400 μm distance from the soma and a standard deviation of 12 μm. Time constants and amplitudes of synaptic conductance changes were varied by multiplying with a random number drawn from a normal distribution with mean 1 and standard deviation 0.3 for time constants and 0.1 for amplitudes. Onset times of synaptic conductance changes were simulated as a Poisson process to yield a mean EPSC frequency of 5 Hz as described by Schmidt-Hieber and Häusser (2013).

## Funding

Christoph Schmidt-Hieber is a Feodor Lynen scholar of the Alexander von Humboldt Foundation and supported by grants from the European Research Council, the Wellcome Trust and the Gatsby Charitable Foundation.

### Conflict of interest statement

The authors declare that the research was conducted in the absence of any commercial or financial relationships that could be construed as a potential conflict of interest.

## Supplementary material

Stimfit is released under the GNU General Public License and is available from http://www.stimfit.org. Parts of the results presented here have been previously published in abstract form (Schlögl et al., 2013). Supplementary code listings accompany this publication.
