# Supplementary material for: Stimfit: quantifying electrophysiological data with Python
Source: Front Neuroinform. 2014 Feb 21;8:16. doi: 10.3389/fninf.2014.00016 (PMC3931263; doi:10.3389/fninf.2014.00016)
Supplement: Supplementary file 1 [file DataSheet1.PDF]

Frontiers in Neuroinformatics

Supplement
14 December
2013

Stimfit: quantifying electrophysiological data with Python

Segundo J Guzman [1], Alois Schlögl [1] and Christoph Schmidt-Hieber [2,3,*]

[1] *Institute of Science and Technology (IST) Austria, Klosterneuburg, Austria*
[2] *Wolfson Institute for Biomedical Research, University College London, London, UK*
[3] *Department of Neuroscience, Physiology and Pharmacology, University College London, London, UK*

Correspondence*:
Christoph Schmidt-Hieber
Wolfson Institute for Biomedical Research, University College London, London
WC1E 6BT, United Kingdom , c.schmidt-hieber@ucl.ac.uk

Python in Neuroscience II

SUPPLEMENTARY MATERIAL

LIST OF SUPPLEMENTARY LISTINGS

1 Python script showing the four layered analysis 2
2 Least squares fitting with Python scripting 3
3 Event detection with Python 4

Supplementary Listing 1. Python script showing the four layered analysis

```
import stf

def syn_conductance(trace):
    """
    calculates the peak conductance (mS) of an evoked
    synaptic response that starts at 10 ms and is acquired at a
    holding potential of -70 mV.
    Arguments:
    trace   -- the zero-base index of the trace in the recording

    Returns:
    the peak conductance in mS. An exception is raised if the trace
    units are not in pA.

    """
    # Layer I: check that a file is open
    if not stf.check_doc():
        raise IOError("File is not open")

    # Layer II: set appropiate cursors
    stf.set_base_start(  0, is_time = True)
    stf.set_base_end(    5, is_time = True)
    stf.set_peak_start( 10, is_time = True)
    stf.set_peak_end(   15, is_time = True)
    stf.set_peak_direction("down")
    stf.set_peak_mean(1)

    # Layer III: move to desired trace
    stf.set_trace(trace)

    if stf.get_yunits() != "pA":
        raise RuntimeError('Trace does not have units in pA')

    # Layer IV: return resistance in mS
    current = ( stf.get_base() - stf.get_peak() )/1000. # current in nA
    return(current/-70.) # conductance in mS
```

Supplementary Listing 2. Least squares fitting with Python scripting

```
import stf

def show_selected_tau():
    """
    Fits the data to a monoexponential function using the least squares
    method. Assumes peak and base cursors are located as in Listing 1.

    Returns:
    a 2 column table with the list of selected traces and the
    corresponding time course. It allows copy/paste in
    spreadsheet programs
    """

    # Layer II: set fit cursors
    stf.set_fit_start( stf.peak_index() ) # start fit cursor at the peak
    stf.set_fit_end( stf.get_size_trace()-1 ) # end fit cursor at the end of trace

    # Layer III: get list of selected traces
    mytraces = stf.get_selected_indices()

    # Layer IV: get tau from monoexponential function in ms
    mytable = {}
    for idx in mytraces:
        stf.set_trace(idx)
        tau = stf.leastsq(fselect = 1, refresh = False)["Tau_0"]
        mytable["Trace %.3d" % idx] = tau

    stf.show_table(mytable) # show results in a table
```

Supplementary Listing 3. Event detection with Python

```
import stf
import numpy as np
import matplotlib.pyplot as plt

def events(template, deconv_th=4.5, matching_th=3.0, deconv_min_int=10.0,
           matching_min_int=100.0):
    """
    Detects events using both deconvolution and template matching. Requires
    an arbitrary template waveform as input. Thresholds and minimal intervals
    between events can be adjusted for both algorithms. Plots cumulative
    distribution functions.
    """

    # Compute criteria
    deconv_amps, deconv_ieis = detect(template, "deconvolution", deconv_th,
                                      deconv_min_int)
    matching_amps, matching_ieis = detect(template, "criterion", matching_th,
                                          matching_min_int)

    fig = plt.figure()

    amps_ax = fig.add_subplot(121)
    amps_ax.hist([deconv_amps, matching_amps], bins=50, cumulative=True,
                 normed=True, histtype="step")
    amps_ax.set_xlabel("Amplitudes (pA)")
    amps_ax.set_ylabel("Cumulative probability")

    ieis_ax = fig.add_subplot(122)
    ieis_ax.hist([deconv_ieis, matching_ieis], bins=50,
                 cumulative=True, normed=True, histtype="step")
    ieis_ax.set_xlabel("Interevent intervals (ms)")
    ieis_ax.set_ylabel("Cumulative probability")

def detect(template, mode, th, min_int):
    """
    Detect events using the given template and the algorithm specified in
    'mode' with a threshold 'th' and a minimal interval of 'min_int' between
    events. Returns amplitudes and interevent intervals.
    """
    # Compute criterium
    crit = stf.template_matching(template, mode=mode, norm=False, lowpass=0.1,
                                 highpass=0.001)

    dt = stf.get_sampling_interval()

    # Find event onset times (corresponding to peaks in criteria)
    onsets_i = stf.peak_detection(crit, th, int(min_int/dt))

    trace = stf.get_trace()

    # Use event onset times to find event amplitudes (negative for epscs)
    peak_window_i = min_int / dt
    amps_i = np.array([int(np.argmin(trace[onset_i:onset_i+peak_window_i])+onset_i)
                       for onset_i in onsets_i], dtype=np.int)

    amps = trace[amps_i]
    ieis = np.diff(onsets_i) * dt

    return amps, ieis
```
